# Impact of Chronic Moderate Psychological Stress on Skin Aging: Exploratory Clinical Study and Cellular Functioning

**DOI:** 10.1111/jocd.16634

**Published:** 2024-11-06

**Authors:** Muriel Pujos, Cécile Chamayou‐Robert, Marine Parat, Magali Bonnet, Sandra Couret, Alessia Robiolo, Olivier Doucet

**Affiliations:** ^1^ COTY Research and Development Monaco Monaco

**Keywords:** cortisol, DNA damage, epinephrine, extracellular matrix, psychological stress, skin aging, skin barrier, wound healing

## Abstract

**Introduction:**

Skin is continuously exposed to environmental external and internal factors, including psychological stress (PS). PS has been reported to trigger different dermatoses such as psoriasis, atopic dermatitis, vitiligo, alopecia areata, and acne through the release of cortisol and epinephrine.

**Objective:**

To clinically explore PS‐induced measurable skin aging signs in subjects with moderate versus mild chronic PS, and to investigate the effect of chronic PS on DNA damage at cellular level.

**Methods:**

In vitro stress tests with cortisol and epinephrine, and with cortisol only on extracellular matrix (ECM) synthesis, as well as on normal human skin fibroblast and keratinocyte functioning, including skin barrier and wound healing were performed.

**Results:**

Moderately stressed subjects in the context of the clinical study had a significantly decreased antioxidant potential and impacted skin barrier integrity, as well as significantly increased signs of microrelief alterations (skin texture and fine lines) reaching an increased severity of about 32.9%. At a cellular level, DNA integrity, ECM synthesis, wound healing, and skin barrier parameters were impacted by increased stress hormone levels.

**Conclusion:**

The clinical exploratory studies presented herewith, as well as the study of cell functioning under stress, have provided evidence that chronic PS significantly affects skin homeostasis and triggers skin aging.

## 1 Introduction

Skin is continuously exposed to external and internal exposome factors, including sun irradiation, tobacco, pollution, and temperature change, as well as nutrition, medication, lack of sleep, and psychological stress (PS); all have been identified to potentially trigger skin aging [1, 2].

PS is a complex process, involving various biological pathways, cells and messengers, leading to a wide range of minor or major health disorders [3, 4, 5, 6, 7]. During exposure to stress, the hypothalamic–pituitary–adrenal axis (HPA) releases mediators, such as cortisol and epinephrine [8, 9, 10]. Like most organs, the skin may be infused with these substances. PS has been reported to potentially trigger cutaneous dermatoses, including psoriasis, atopic dermatitis, the pathological impairment of barrier function, and wound healing [11]. It may also contribute to immune dysfunction [12, 13].

Previous studies have demonstrated that PS can cause alterations in the permeability of skin barrier homeostasis under specific conditions, mediated by increased endogenous glucocorticoids which alter both barrier homeostasis and stratum corneum integrity [14, 15].

In spite of the known impact of PS on several skin disorders, its different molecular mechanisms regarding human skin have not been extensively studied. One study suggested a link between psychological status and skin barrier permeability in humans presenting specific skin conditions and studied the impact of stress‐induced derangements in epidermal function as precipitators of inflammatory dermatoses [16].

Despite published information regarding the impact of several biological markers of PS on immune function and inflammatory markers, only little is known about how exactly this may cause a visible impact on facial aging [17, 18, 19]. Currently, only one publication refers to visible signs of aging due to PS. This study was conducted in subjects exposed to occupational stress induced by a specific type of professional activity in very specific conditions [20].

The global aim of the present investigations was to evaluate the measurable changes in the skin that anyone could encounter in their life due to moderate chronic PS, through a clinical exploratory study.

To this end, we evaluated the modifications that may result in skin aging signs in moderately stressed subjects compared to mildly stressed subjects through the assessment of the antioxidant capacity, barrier integrity, and roughness of the skin.

Moreover, the different potential mechanisms of the action of PS were assessed at cellular level through the impact of cortisol and epinephrine on normal human skin keratinocyte and fibroblast functioning, as both play an important role in the skin composition. In vitro skin aging biomarker assays included DNA damage, ECM gene expression, wound healing, and skin barrier integrity.

## 2 Material and Methods

### 2.1 Clinical Exploratory Study

The clinical exploratory study assessed PS on clinical skin aging markers through instrumental measurements.

#### 2.1.1 Settings

This non‐interventional study was approved by an independent ethics committee.

All subjects provided written informed consent prior to inclusion. The study respected Good Clinical Practices and adhered to the principles of the Declaration of Helsinki.

#### 2.1.2 Subjects

Forty women aged between 35 and 55 years exposed to mild (*n* = 20) and moderate (*n* = 20) chronic psychological stress according to the “Perceived Stress Scale” questionnaire were recruited [21]. Chronic PS was defined as a consistent sensation of being pressured and overwhelmed over a prolonged time period.

All participants had to be nonsmokers, not consuming alcohol, working or staying indoor, and were good sleepers and not overexposing themselves to the sun (neither sun worshippers nor sun lamp users). Moreover, all subjects were to be on a standard diet and were not under caloric restrictions or other particular dietary regimens.

#### 2.1.3 Assessments

The total antioxidant capacity of the skin was measured using the FRAP assay on skin stripping samples [22]. Skin samples were made using Corneofix foils (Courage‐Khazaka, Germany).

Skin surface texture parameters included the assessment of “crow's feet” using a real 3D microtopography imaging system (Primoslite GFMesstechnik GmbH, Germany). The skin surface was reconstructed using an algorithm to generate 3D images. The following parameters were measured: R3z (initial roughness), Rt (total roughness), Rp (profile peak height), RSm (roughness amplitude mean), and S (peak amplitude mean).

Transepidermal water loss (TEWL) was assessed using a Tewameter 300 (Courage + Khazaka, electronic GmbH).

#### 2.1.4 Statistical Analysis

Quantitative variables were described by means and standard deviations. A significance level of *p* < 0.05 was considered as statistically significant.

All calculations were made using a Microsoft Excel 2013 (version 15.0.4893.1002; Microsoft, USA) worksheet running on Microsoft Windows 8.1 Professional (Microsoft, USA).

Data were submitted to the two‐way Student's *t*‐test for paired data, or to the Mann–Whitney *U*‐test based on data normality assumption. Statistical analysis was carried out using NCSS 10 professional (version 10.0.12; NCSS 10 Statistical Software, NCSS, LLC. Kaysville, Utah, USA) running on Microsoft Windows Server 2008 R2 Standard (Microsoft, USA).

#### 2.1.5 In Vitro Assays

In vitro assays were conducted on a molecular level to assess the impact of cortisol and epinephrine on skin aging markers.

#### 2.1.6 Cell Culture

Normal human keratinocytes (Biopredic International, France) were maintained in “keratinocytes‐SFM” medium supplemented with bovine pituitary extract (30 μg/mL) and recombinant epidermic growth factor (rEGF, 0.2 ng/mL). Normal human fibroblasts (Promocell Human Centered Science, Germany) were maintained in DMEM medium supplemented with fetal bovine serum 10%. Cultures were performed in a 37°C humidified incubator with 5% CO_2_ atmosphere.

Reconstructed human epidermis (RHE, Episkin Laboratories, Lyon, France) was cultured in growth medium at 37°C in a humidified 5% CO_2_ atmosphere.

#### 2.1.7 DNA Damage

The standard alkaline comet assay was performed as described by Singh et al. [23] A modified protocol was used to improve the sensitivity of the assay by using a specific enzyme, formamidopyrimidine DNA glycosylase (FPG). This enzyme allows the detection of 8‐oxoguanine and other purine oxidation products formed when cell nuclei are exposed to oxidative stress and which specifically recognizes oxidized DNA bases to convert them into strand breaks [24].

Normal human keratinocytes and fibroblasts were treated with cortisol or epinephrine solutions for 24 h. Cells were then trypsinized, embedded in agarose, and deposited onto microscope slides. After a bath in the lysis solution, slides were washed with BSA enzymatic buffer and treated with FPG for 30 min at 37°C. Electrophoresis was carried out for 20 min at 25 V and 300 mA. Slides were washed with Tris buffer (pH 7.5), dehydrated in 100% methanol and dried.

Each slide was stained with ethidium bromide and examined at 250× magnification using a fluorescence microscope (Olympus Optical Co., Tokyo, Japan) equipped with a U‐MWG2 dichroic mirror (band‐pass filter, 510–550 nm; long‐pass filter, 590 nm). Image analysis was performed using the Komet software (version 6.0 Andor Technology, Belfast, Northern Ireland). A total of 50 randomly selected cells were analyzed per slide using Fenestra Komet 6.0 image analysis software (Andor Technology, Belfast, Northern Ireland). DNA damage was expressed as the Olive Tail Moment (OTM; arbitrary units); 100 OTM values were determined for each sample, 50 from each of two separate slides.

OTM values were distributed into classes between the minimal and maximal values. A non‐linear regression analysis was performed on the OTM distribution frequencies by using a chi‐squared function with TableCurve 2D software (version 5.0; Jandel Scientific Software, San Rafael, CA). The calculated degrees of freedom (*n*) for this function were quantitative measures of the DNA damage for a sample [25]. The n was termed “chi‐2 OTM,” and was used as the sole parameter for assessing levels of DNA damage. The significance of the differences between chi‐2 OTM values of non‐treated cells and those treated with cortisol or epinephrine was analyzed using the Student's *t*‐test.

#### 2.1.8 Extracellular Matrix Synthesis

Normal human skin fibroblasts were cultured with cortisol (Sigma Aldrich, Merck KGaA, Darmstadt, Germany) at 0.1, and 1 μM added to the culture medium (DMEM) for 48 h. Total RNA was extracted with a Pure RNA Tissue kit (Thermofisher, Strasbourg, France) and quantified with a spectrophotometer at 260 nm. First‐strand cDNA was then synthesized by using a High‐Capacity cDNA Reverse Transcription kit. Real‐Time RT‐PCR reactions were carried out with the 7300 Real Time PCR System using the TaqMan primers and probes (Applied Biosystems, Waltham, Massachusetts, États‐Unis) specific to each gene. Relative changes in gene expression (RQ) were calculated according to the 2^−ΔΔCT^ method, utilizing the 18S RNA as housekeeping gene. Results were compared using a Student's *t*‐test; a *p* < 0.05 was considered statistically significant.

#### 2.1.9 Wound Healing

Normal human skin fibroblasts and keratinocytes were seeded in a 35‐mm petri dish containing culture‐insert and incubated at 37°C with CO_2_ 5% and an appropriate culture medium to obtain a confluent monolayer. To inhibit cell proliferation, mitomycin C was added for 2 h and removed by washing with phosphate buffered saline (PBS) solution. The culture‐insert was removed from the petri dish. Cells were cultured with cortisol (Sigma Aldrich, Merck KGaA, Darmstadt, Germany) at 0.1 and 1 μM added to the culture medium. During incubation, cell culture images were regularly recorded using a real‐time monitoring of cell culture (Cytonote, Iprasense, France) connected to Horus software (iPrasense, France). The wound area (cell‐free area) was measured, and the rate of cell migration was calculated [(wound area at *t* = 0—wound area at *t* = end) / time of the duration of migration].

#### 2.1.10 Skin Barrier Integrity

Reconstructed human epidermis (RHE) was cultured with growth medium containing cortisol at 2.5 or 5 μM for 8 days, the medium was changed every day. At the end of treatment, RHE was fixed in buffered formaldehyde 4% and embedded in paraffin. Sections were de‐paraffinized in xylene and rehydrated in a series of graded alcohols, then antigen retrieval was carried out in a citrate buffer and non‐specific sites were blocked with goat serum. Sections were rinsed with PBS solution and incubated with specific primary antibodies (Filaggrin Ref: ab218395, Abcam; Loricrin ref.: ab85679, Abcam, Cambridge, UK). The sections were washed with PBS‐Tween and the secondary antibody, AlexaFluor 488 and AlexiaFluor 350, was applied. Nuclei were stained with propidium iodide (Sigma Aldrich, Merck KGaA, Darmstadt, Germany). Sections were then mounted in Gel Mount medium. The surface of filaggrin and loricrin staining was quantified with an image analysis software (ZEN 2 blue edition, Zeiss, Jena, Germany) and normalized with the total surface of the RHE.

## 3 Results

### 3.1 Clinical Exploratory Study

Overall, 36 subjects completed the study, 18 women with mild PS and 18 with moderate PS. The subjects were aged 50.2 ± 0.9 and 49.7 ± 1.1 years in both mild and moderate PPS groups, respectively. PSS scores were significantly (*p* = 0.001) lower in the mild stress group (12.0 ± 0.4) than in the moderate stress group (23.1 ± 0.6), confirming the difference between both groups.

Results from the FRAP assay showed that women suffering from moderate stress had a significantly lower (119.7 ± 7.5 μMFe^2+^; mean difference: 12.2%; *p* = 0.04) antioxidant capacity than those suffering from mild stress (163.3 ± 5.5 μMFe^2+^).

A statistical difference (all *p* < 0.05) between both panels of women was observed for all roughness parameters (Table 1). A 32.9% increase of the Rp profile peak height confirms a change in the microrelief, characterized by an increase of fine lines and texture alteration in moderately stressed vs. mildly stressed subjects.

**TABLE 1:** Mean values for skin surface texture parameters in mild or moderately stress subjects.

Parameter	Mild PS (*n* = 18)	Moderate PS (*n* = 18)	*p* ^a^
R_max_, maximal roughness (μm)	467.73 ± 25.12	550.16 ± 37.12 (+17.6%)	0.0373
R3z, initial roughness (μm)	130.95 ± 5.42	145.36 ± 6.37 (+11.0%)	0.0471
Rt, total roughness (μm)	0.5018 ± 0.0268	0.5893 ± 0.0393 (+17.4%)	0.0372
Rp, profile peak height (μm)	207.15 ± 13.76	275.23 ± 20.32 (+32.9%)	0.0045
RSm, roughness amplitude (μm)	1.69 ± 0.05	1.88 ± 0.08 (+11.2%)	0.0274
S, peaks amplitude (μm)	442.20 ± 6.26	460.41 ± 4.47 (+4.1%)	0.0119

*Note:* All skin surface texture parameters were significantly more impacted by moderate than by mild PS.

^a^ *t*‐test.

A statistically significant difference (14.4% *p* < 0.033) of mean TEWL values between both panels was observed with a higher TEWL (12.4 g/h/m^2^) in the moderate compared to the mildly stressed panel (10.8 g/h/m^2^).

### 3.2 In Vitro Assays

#### 3.2.1 DNA Damage

Cortisol treatment generated a dose‐dependent increase of DNA oxidative damage, such as 8‐oxoguanine. Significant damage was observed with the 1 μM dose (OTM median = 2.27, Chi^2^OTM = 3.83, *p* < 0.001) on keratinocytes, and with 0.5 μM on fibroblasts (OTM median = 1.67, Chi^2^OTM = 3.17, *p* < 0.001).

Epinephrine‐induced significant DNA damage, both on keratinocytes and fibroblasts, with a dose of 0.5 μM (OTM median = 1.08, Chi^2^OTM = 2.59, *p* < 0.001) and of 1 μM (OTM median = 1.23, Chi^2^OTM = 3.08, *p* < 0.001), respectively.

**FIGURE 1:**
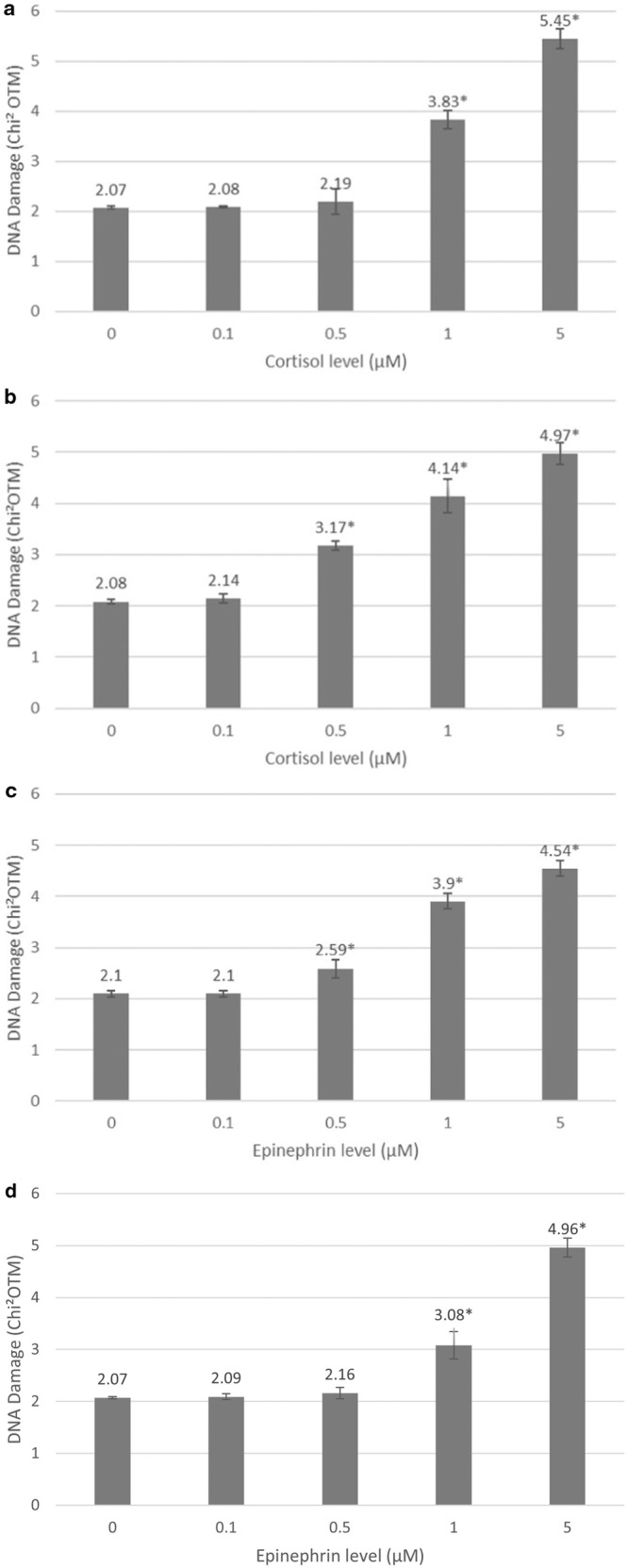
DNA damage (mean±SEM, n=100). Cortisol‐induced oxidative lesions on (a) normal human keratinocytes and (b) normal human fibroblasts. Epinephrine‐induced oxidative lesions on (c) normal human keratinocytes and (d) normal human fibroblasts. *Significant compared to non‐stressed cells (p < 0.001).

Results are given in Figure 1.

#### 3.2.2 Extracellular Matrix Synthesis

**FIGURE 2:**
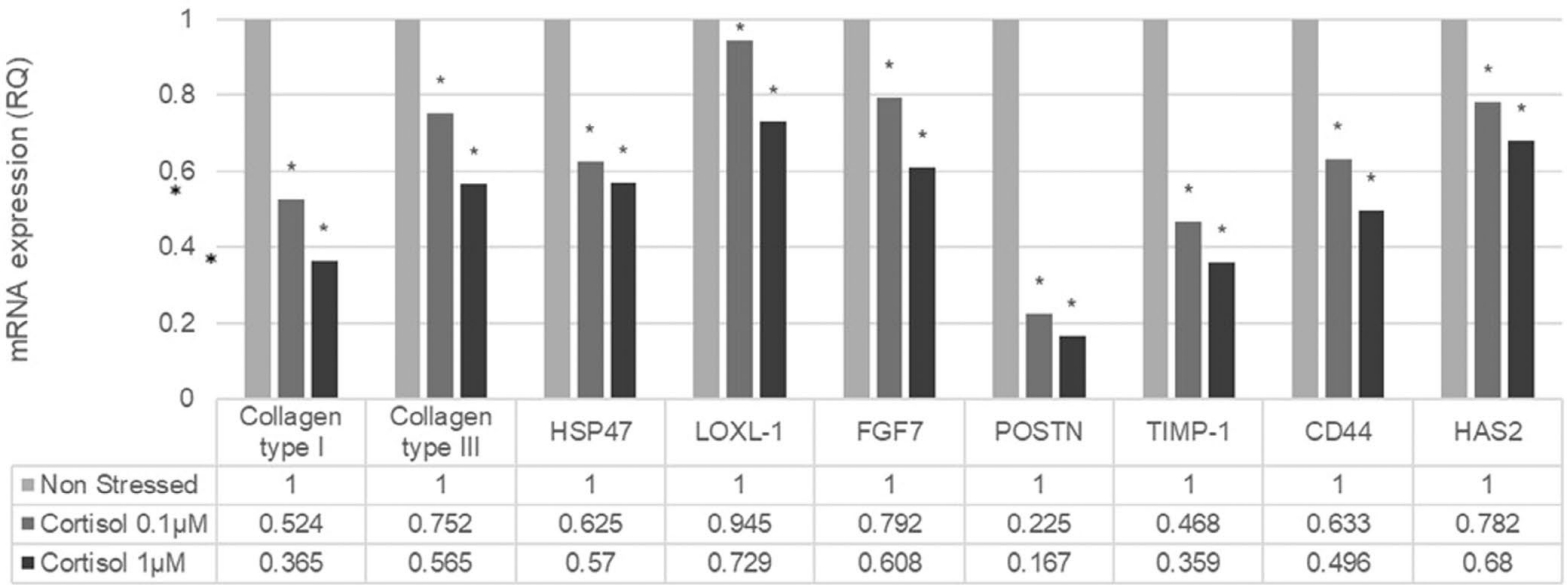
Extracellular matrix gene expression on normal human fibroblasts treated with cortisol at 0.1 and 1 μM (mean ± SEM, *n* = 5). *Significantly different to non‐stressed cells (*p* < 0.05).

Collagen type I, collagen type III, and HSP47 were significantly (*p* < 0.05) downregulated, with the two different doses of cortisol, as well as the tissue inhibitor of metalloproteins, TIMP1. LOXL1 mRNA was downregulated by 27% with the highest cortisol dose, predicting a possible impairment of the skin elastin network. The expression of two genes related to hyaluronic acid content, HAS2 and CD44, were downregulated (Figure 2).

A significant (*p* < 0.05) downregulation of fibroblast growth factor 7 (FGF7) and periostin (POSTN) gene expression was observed with a decrease of about 80% for POSTN (Figure 2).

#### 3.2.3 Wound Healing Impairment

**FIGURE 3:**
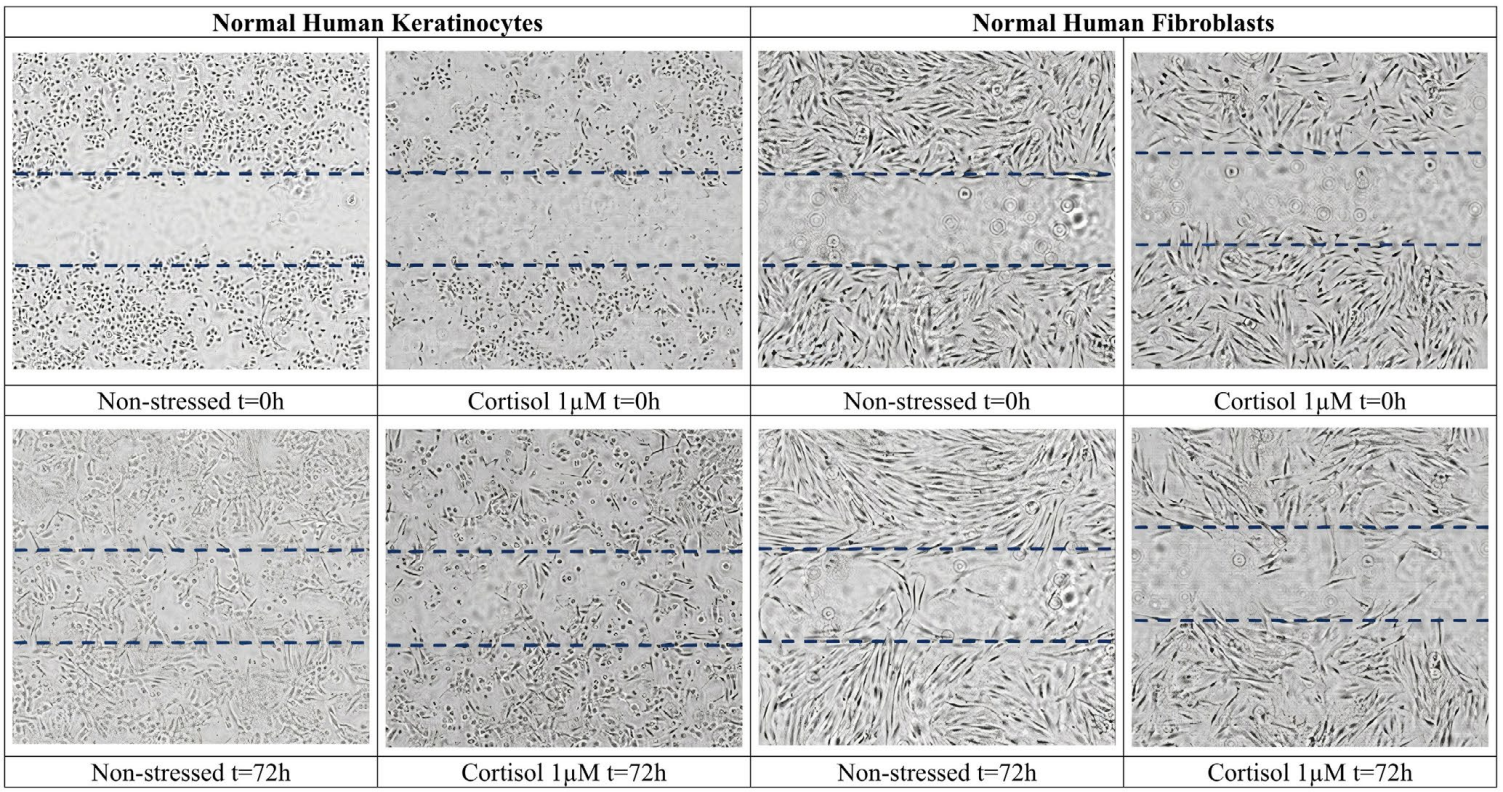
Normal Human Keratinocytes and Normal Human Fibroblasts migration under Cortisol‐induced stress versus Non‐stressed cells. [Correction added on 23 November 2024, after first online publication: Figure 3 has been updated in this version.]

Cortisol‐induced stress impaired both the speed of wound healing and the cell migration ability of keratinocytes. As shown in Figure 3, the wound area (cell‐free area) was almost totally closed after 72 h for non‐stressed keratinocytes (wound area = 6%), whereas wound areas were larger for cells exposed to cortisol 0.1 μM (wound area = 13%) and 1 μM (wound area = 28%). Similarly, the cell migration rate of keratinocytes in “stressed skin” decreased by up to 19% with a cortisol dose of 1 μM. In normal human fibroblasts, the wound area in samples of non‐stressed cells was 55% (Figure 3) reaching 86% in the presence of cortisol at 1 μM after 72 h. The cell migration rate significantly (p < 0.05) decreased by 73%.

#### 3.2.4 Skin Barrier Damage

**FIGURE 4:**
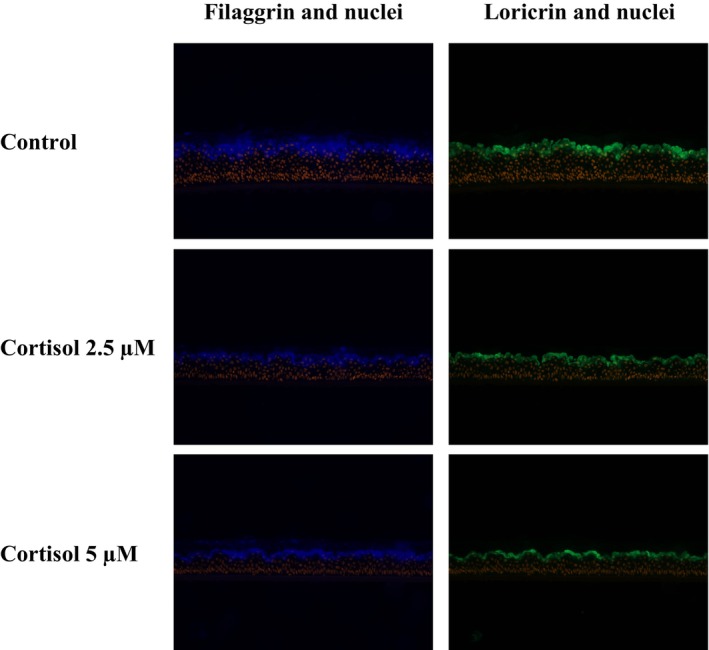
Filaggrin and Loricrin immunostaining on Reconstituted Human Epidermis submitted to Cortisol‐induced stress at 2.5µM & 5µM.

**FIGURE 5:**
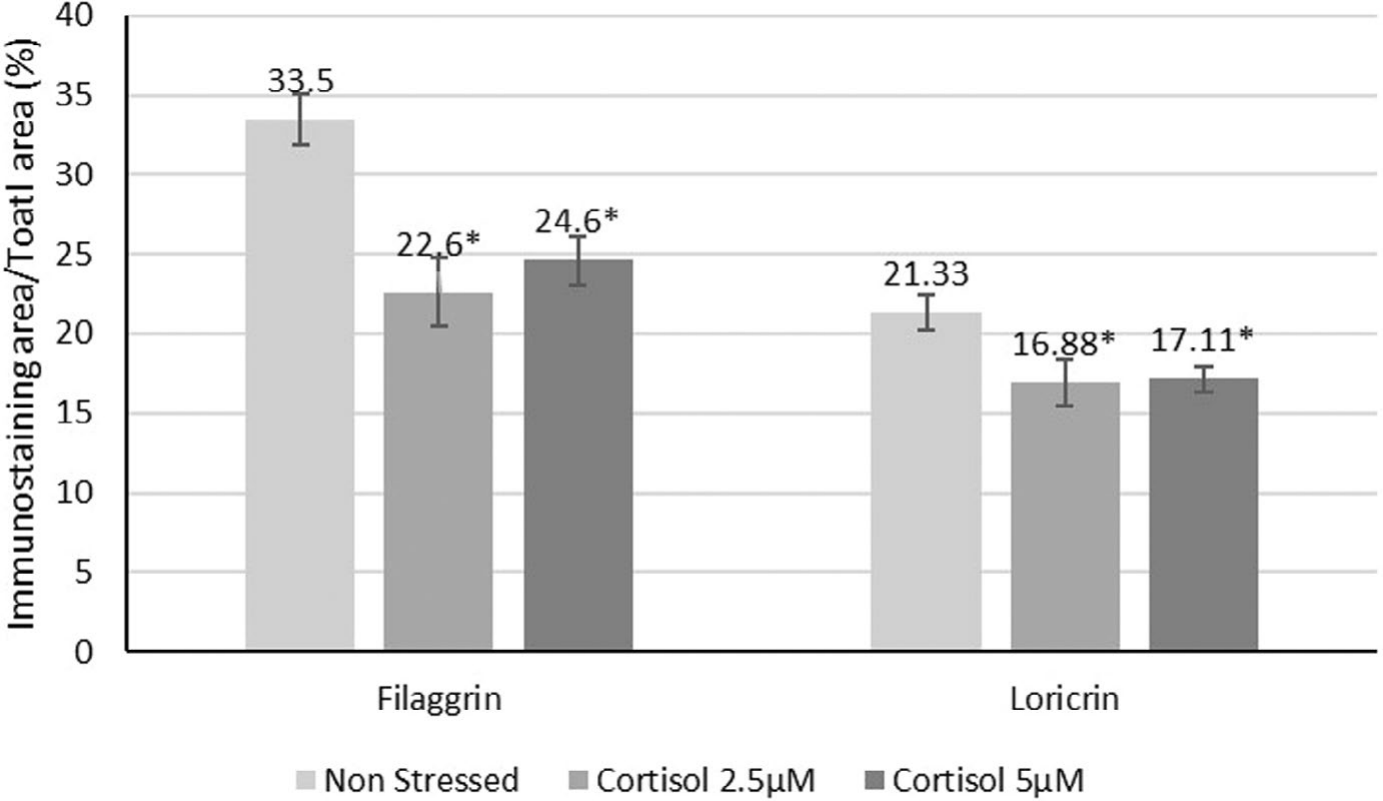
Quantification of filaggrin and loricrin on RHE treated with cortisol (mean ± SEM, *n* = 3). *Significant compared to non‐stressed cells (*p* < 0.05).

RHE cultured in normal conditions not exposed to cortisol showed a clear staining of filaggrin and loricrin expressed in the granular layer (Figure 4). In the presence of cortisol, the filaggrin synthesis significantly decreased by 32% and 26% with cortisol 2.5 and 5 μM, respectively. A decrease of 20% of loricrin was also observed with the highest cortisol dose. See Figure 5 for complete results.

## 4 Discussion

This work confirms the impact of chronic moderate PS on skin aging markers including antioxidation capacities, skin texture, and barrier integrity.

This work also provides evidence that, on a cellular level, stress “hormones” such as cortisol and epinephrine, significantly impact wound healing and DNA integrity, as well as the ECM synthesis and alter the synthesis of the epidermal barrier.

According to the FRAP assay, subjects exposed to moderate PS had a significantly (*p* = 0.04) lower antioxidant capacity than those exposed to mild PS, with a mean percent difference of 12%. Furthermore, moderately stressed subjects had significantly increased skin roughness parameters compared to mildly stressed subjects, translating into a significant increase in fine lines and roughness in 32.9% of the moderate stress group. Both skin relief alteration and decreased antioxidant potential parameters can contribute significantly to the first visible signs of aging.

The impact of moderate PS on the skin barrier integrity was demonstrated with an increased TEWL in moderately stressed subjects, as previously reported by Schoenfelder et al. [26]. This increased TEWL translates into a weaker skin barrier which is more susceptible to the impact of external exposome factors on the skin homeostasis.

The cellular assays confirm that increased cortisol and epinephrine levels, both biomarkers of PS, have a deleterious impact on skin functioning, in both skin keratinocytes and fibroblasts. The wound healing process was slowed down, with a significant downregulation of fibroblast growth factor 7 (FGF7) and an 80% decrease of periostin (POSTN) gene expression. DNA damage significantly (*p* < 0.001) increased with increasing cortisol and epinephrine doses. Moreover, collagen type I, collagen type III, and HSP47 were significantly downregulated. So was the tissue inhibitor of metalloproteins, TIMP1 and LOXL1 mRNA, predicting a possible impairment of the skin elastin network, while HAS2 and CD44—both related to hyaluronic acid content—decreased. Furthermore, in the presence of cortisol, the filaggrin synthesis was significantly reduced by 32% and 26% with cortisol doses of 2.5 μM and 5 μM respectively, and loricin decreased by 20% after exposure to the highest cortisol dose, confirming that cortisol impacts the skin integrity. These results support the hypothesis that PS, known for causing the release of cortisol and epinephrine, impairs skin homeostasis and probably contributes to premature skin aging [2]. This was already shown in 2020 by De Tollenaere *et al*., who assessed skin homeostasis under multi‐stressed conditions [27]. Other previous work has reported that, after local activation, cortisol is expressed in the epidermis and in dermal fibroblasts, confirming our findings regarding the relationship between cortisol, skin components, cell proliferation, wound healing, inflammation, and aging [28].

The study opened a new perspective in considering chronic PS as a skin aging exposome, in combination with the performed in vitro investigation [2, 9, 29].

In conclusion, chronic PS affects the skin biology through different pathways: from DNA damage to the alteration of gene expression, translating into the alteration of the skin microrelief and ultimately in wrinkles, in the impairment of the natural antioxidant barrier and the natural physical skin barrier. PS has become an increasingly important issue in our daily life. It not only impacts our mental well‐being but also influences our physical integrity, including that of the skin. The present investigations confirm that chronic PS impacts skin appearance through stress hormones such as cortisol and epinephrine, and these studies have provided the first supporting elements. Further research and clinical studies need to be conducted to confirm these findings.

## Author Contributions

Magali Bonnet, Sandra Couret, and Alessia Robiolo performed the technical investigation. Marine Parat, Cecile Chamayou‐Robert, and Muriel Pujos designed the studies and analyzed and interpretated the results. Muriel Pujos and Cecile Chamayou‐Robert wrote the manuscript. Muriel Pujos, Cecile Chamayou‐Robert, Marine Parat, and Olivier Doucet reviewed the manuscript.

## Ethics Statement

This non‐interventional study was approved by an Independent Ethical Committee for Non‐Pharmacological Clinical trials (ref. 2018/04, Società Scientifica Italiana per le Indagini Cliniche Non Farmacologiche Via XX Settembre 30/4 – 16121 Genova). The study respected Good Clinical Practices and adhered to the principles of the Declaration of Helsinki. All subjects provided written informed consent prior to inclusion.

## Conflicts of Interest

The authors are employees of Coty Inc., Monaco.

## Data Availability

The data that support the findings of this work are available upon reasonable request from the corresponding author.
